# Prenatal Stress as a Risk Factor for Maternal–Foetal Morbidity: A Longitudinal Study

**DOI:** 10.3390/healthcare12030312

**Published:** 2024-01-25

**Authors:** Rocío Palomo-Gómez, Azahara Rúger-Navarrete, Irene Antúnez-Calvente, Juana María Vázquez-Lara, Luciano Rodríguez-Díaz, Juan Gómez-Salgado, Francisco Javier Riesco-González, María Dolores Vázquez-Lara, Francisco Javier Muñoz-Vela, Francisco Javier Fernández-Carrasco

**Affiliations:** 1 Department of Obstetrics, Hospital of La Línea de la Concepción, 11300 Cadiz, Spain; 2 Department of Nursing, Faculty of Health Sciences of Ceuta, University of Granada, 51001 Ceuta, Spainjavier.fernandez@ugr.es (F.J.F.-C.); 3 Department of Obstetrics, Hospital Universitario Germans Trias i Pujol, 08916 Badalona, Spain; 4 Department of Sociology, Social Work and Public Health, Faculty of Labour Sciences, University of Huelva, 21007 Huelva, Spain; 5 Safety and Health Postgraduate Programme, Universidad Espíritu Santo, Guayaquil 092301, Ecuador; 6 Department of Obstetrics, Hospital Universitario Punta de Europa, 11207 Algeciras, Spain; 7 Department of Nursing, Menendez Tolosa Health Center, Andalusian Health Service, 11202 Algeciras, Spain; mdolores.vazquez.sspa@juntadeandalucia.es; 8 Department of Nursing, Faculty of Health Sciences, University of Málaga, 29701 Málaga, Spain

**Keywords:** prenatal stress, pregnancy, cortisol, maternal morbidity, pregnancy complications, maternal physiology, coping with stress

## Abstract

Pregnancy is one of the most complex periods in a woman’s life, not only because of the biological changes involved but also because of the psychological aspects. Stress during pregnancy refers to the concerns and distress that arise during pregnancy and that can be assessed by means of psychological and physiological scales. The aim of this study was to analyse prenatal stress and to evaluate its consequences on the health of both the mother and the foetus. A descriptive longitudinal study was carried out on a sample of 398 pregnant women being followed up during their entire pregnancy, who gave birth at the Punta de Europa University Hospital in Algeciras (Spain) between September 2021 and August 2023. The Prenatal Distress Questionnaire (PDQ) was used, as well as serum cortisol levels in each trimester of pregnancy and birth experience using the Childbirth Experience Questionnaire in its validated Spanish version, CEQ-E. Demographic and obstetric variables were included. One of the main findings was that experiencing more stress in late pregnancy had a negative impact on obstetric outcomes. Women who had higher levels of prenatal distress had higher blood cortisol levels and increased risk of having a caesarean section at delivery. A significant negative correlation was also found between stress and Apgar test values in the first minute of life. It is concluded that interventions promoted by the health system that provide comprehensive prenatal care contribute to decreased stress as perceived by these pregnant women, thus reducing the risk of maternal and foetal morbidity.

## 1. Introduction

Pregnancy is one of the most complex stages in a woman’s life and one that is full of change [1]. From a psychological point of view, a woman’s way of thinking and feeling, and her lifestyle have to be modified and adjusted to the process of becoming a parent [2,3,4], while she actively acquires skills in her role as a mother. The whole process can be very stressful for the woman [5,6] who needs a great deal of emotional support at this stage of life [7].

On the other hand, from a physiological point of view, there is a relationship between the duration of pregnancy, the ability to carry the foetus to term, and serum levels of cortisol, a hormone that increases the levels of anxiety and stress in the mother [7].

In recent years, the relationship between the hypothalamic–pituitary–adrenal (HPA) system and stress and its effects on the foetus have been studied. Doktorchiky and Premji [8] indicated that increased levels of anxiety during pregnancy were associated with an increased risk of preterm birth. Prenatal maternal distress has also been associated with reduced anthropometric measurements at birth [9].

The HPA axis is activated in acute stress situations by the adrenocorticotropic hormone (ACTH) [10], secreted at the pituitary level as a result of central corticotropin-releasing hormone (CRH) and arginine vasopressin (AVP) stimulation, which ultimately leads to the secretion of glucocorticoids, mainly cortisol, in the adrenal glands. Cortisol secretion has a circadian and episodic rhythm throughout the 24 h, peaking at about waking time and decreasing to minimum levels during the first hours of sleep [10,11,12,13,14].

This rhythm can be altered by changes in sleep timing (sleep/wake cycle), but only if the disturbance persists for several days [15]. Exposure to light (light/dark cycles), diet, physical activity, stress, and drug intake can influence this cycle [10,11,12,13,14]. Increased or decreased cortisol values can also be found in various physiological situations, such as pregnancy and pathological conditions [13].

For plasma cortisol testing, it is recommended that plasma cortisol be drawn between 8 and 9 o’clock in order to reduce the variability caused by these circadian changes [13]. There are no established normative daily cortisol values during pregnancy [16], but a normal range is considered to be between 5 and 25 µg/dL until the beginning of the second trimester [17] and 2–3 times higher in the third trimester [18].

### Hypothalamic–Pituitary–Adrenal Axis in Pregnancy and Stress

From the first trimester of gestation, CRH production in the placenta, decidua, and foetal membranes significantly increases maternal plasma levels [19].

Maternal pituitary ACTH secretion and plasma ACTH levels are also increased but remain within normal limits, paralleled by increased plasma cortisol levels, with diurnal variation maintained. The maternal adrenal glands gradually become hypertrophic, which physiologically leads to a period of hypercortisolism [20].

Maternal stress may negatively affect the normal functioning of both the maternal and foetal HPA axis, which could lead to negative health consequences for both [21]. Additionally, sustained endogenous foetal cortisol production leads to excessive serum glucocorticoid levels in the foetus, associated with intrauterine growth restriction [22]. Reduced anthropometric measurements and birth weight, as well as increased prematurity, have been related [23,24] to high levels of maternal cortisol and subjective stress in pregnancy [24].

11-beta hydroxysteroid dehydrogenase placental enzyme (HSD-11β) partially protects the foetus by interacting with cortisol; however, studies show that chronic stress can alter the maternal–foetal HPA axis [16,17], meaning that active cortisol could be able to reach the foetal circulation [25]. This, in turn, stimulates the release of CRH from the placenta. Significantly high levels of maternal and umbilical cord CRH have been observed in preterm termination of pregnancies. For this reason, plasma CRH levels have been used as predictors of preterm birth [26,27,28], with levels higher than 90 pM at 26 weeks’ gestation having a predictive value of over 45% for preterm delivery. Besides, foetal growth could be affected by maternal stress due to an excess of sympathoadrenal activation, which would decrease uteroplacental perfusion [27,29].

It has been proven that maternal stress during pregnancy can negatively influence the foetus, both in its physiological and behavioural development, which may affect health and the development of future diseases [30]. However, currently, most clinical practice guidelines and a number of studies on the care of pregnant women do not include the assessment of maternal stress, thus overlooking the opportunity to prevent and treat this problem. In this context, the aim of this study was to correlate prenatal stress with maternal–foetal morbidity during pregnancy and delivery using blood cortisol levels.

## 2. Materials and Methods

### 2.1. Study Design

A descriptive, longitudinal study (1st, 2nd, and 3rd trimesters of pregnancy) was designed. The study variables were ‘subjective stress’ using the Prenatal Distress Questionnaire (PDQ) score [31], serum cortisol levels in each trimester of pregnancy, and birth experience using the validated Spanish version of the CEQ-E Childbirth Experience Questionnaire [32].

In addition, demographic variables such as age, marital status, level of education and income, person providing emotional support, and perceived degree of support were also recorded. Finally, obstetric variables were included, such as having attended childbirth preparation courses, analgesia received for delivery, type of delivery, stitches received, weight, and Apgar test score of the newborn at 1 and 5 min of life.

### 2.2. Population and Sample

The study population consisted of a total of 2562 pregnant women under follow-up who gave birth in a public hospital in Andalusia, the Punta de Europa University Hospital in Algeciras (Cadiz, Spain), between September 2021 and August 2023.

Setting a confidence level of 95% and with a maximum error of 5%, the optimal sample had to be made up of at least 335 subjects. Finally, a total of 398 subjects were recruited to account for possible losses, estimated at around 20%.

The sample was selected by consecutive random probability sampling from women who attended the centre for pregnancy monitoring, met the inclusion criteria, and wished to participate in the study on a voluntary basis.

The inclusion criteria were pregnant women who attended the pregnancy control consultation at the study centre from the first weeks of pregnancy until delivery and completed the gestational health follow-up. 

The following exclusion criteria were applied: pregnant women who suffered from previous pathologies or pathologies originating during pregnancy such as gestational diabetes, premature placental abruption, pre-eclampsia, acute foetal distress, or umbilical cord prolapse; also, the presence of linguistic difficulties, in the case of pregnant women who were unable to write, read, or speak Spanish. 

### 2.3. Instruments

For data collection, two validated and cross-culturally adapted questionnaires were used: the Prenatal Distress Questionnaire (PDQ) [31] and the validated Spanish version of the Childbirth Experience Questionnaire (CEQ-E) [32]. In addition to these two tools, sociodemographic data were collected from the patients.

The PDQ includes a 12-item scale that measures pregnancy distress. It is a way to measure prenatal stress as expressed subjectively by women and specifically during pregnancy. Participants express the level of distress using the following categorical values: 0, not at all; 1, a little; 2, moderately; 3, a lot; and 4, extremely. The total score is the sum of the responses. The higher the score, the more distressed or stressed the pregnant woman is. Cronbach’s alpha score for this scale was 0.71. All scores (ranging from 0–48 points) were aggregated to quantitatively determine prenatal distress.

The CEQ-E contains 22 items relating to the experience of childbirth. Responses to the first 19 items are rated on a 4-point Likert scale (1, strongly agree; 2, mostly agree; 3, mostly disagree; 4, strongly disagree) and the last three items are rated on a visual analogue scale, i.e., pain memory, perceived safety, and control.

The CEQ-E questionnaire contains 4 domains: ‘own capacity’ (8 items related to the sense of control, personal feelings, and pain during childbirth); ‘professional support’ (5 items on professional care); ‘perceived safety’ (6 items on the sense of safety and memories from childbirth); and ‘participation’ (3 items related to having a say in one’s own possibilities to influence position, movements, and pain relief during labour and birth). The internal consistency reliability of the CEQ-E was fair for the overall scale (0.88) and for all subscales (0.80, 0.90, 0.76, 0.68 for ‘own capacity’, ‘professional support’, ‘perceived safety’ and ‘participation’, respectively), values analogous to those of the original version.

### 2.4. Data Collection

Data were collected between 1 September 2021 and 31 August 2023. Contacts were obtained from the retrospective patient management database of the Andalusian Public Health System (Spain).

Once the pregnant woman had been selected, a member of the research team contacted her by telephone to inform her of the characteristics of the study and to offer her to participate in it. Once her consent was obtained, a link to the Google forms was sent to her via WhatsApp so that she could access the virtual questionnaire securely and easily from her own phone.

**Figure 1:**
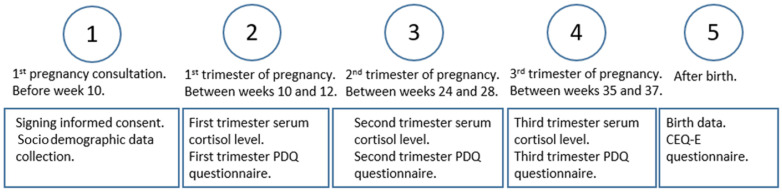
Chronological order of data collection.

An automatic system was set up to obtain the serum cortisol figures for each trimester of pregnancy in such a way that all the gestational tests carried out during the study period were associated with the determination of serum cortisol levels. Cortisol-level data were collected from the Andalusian Health Service database. This way, obtaining these values for each of the participants could be done by just accessing each of their test reports from the hospital laboratory service. Figure 1 explains how the data collection was done chronologically.

### 2.5. Data Analysis

Categorical variables were defined in absolute numbers and percentages. The descriptive analysis of the quantitative variables was carried out using measures of central tendency and dispersion. 

After checking for normality, the nonparametric Mann–Whitney U-test and Kruskal–Wallis test were used in the bivariate statistical analysis to compare quantitative variables. The effect size was calculated using the Hedges’ g test. In addition, testing the assumption of equal variances was addressed using Welch’s *t*-test.

Finally, 2 binary logistic regression models were used to establish associations between the different independent variables and the two dependent variables (PDQ and serum cortisol in the third trimester). 

To be able to use the PDQ score in the third trimester as a dependent variable, it was recorded dichotomously. For this purpose, scores below the mean value were coded as persons with low subjective stress, and scores above the mean value were coded as persons with high subjective stress.

The third-trimester serum cortisol variable was recorded in the same way. Serum cortisol values below the mean value were coded as low stress and serum cortisol values above the mean value were coded as high stress.

To develop this exploratory analysis, confidence intervals (CI) at 95% and a significance level of *p* < 0.05 were considered and achieved. The statistical study was performed using IBM SPSS Statistics version 28.

### 2.6. Ethical Aspects

The general principles contained in the Declaration of Helsinki, updated in 2013 in Fortaleza (Brazil), were considered throughout this study by the entire research team. In addition, the provisions of the current Spanish legislation on biomedical research (Law of 3 July on Biomedical Research) and Law 41/2002 of 14 November on patient autonomy and rights and obligations regarding clinical information and documentation were followed. All personal data were protected in accordance with Organic Law 3/2018, of 5 December, on Personal Data Protection and guarantee of digital rights and in compliance with the General Data Regulation. Permission to conduct this study was obtained from the Andalusian Biomedical Research Ethics Committee on 17 December 2021 (code 2476-N-21).

## 3. Results

### 3.1. Descriptive Analysis

**Figure 2:**
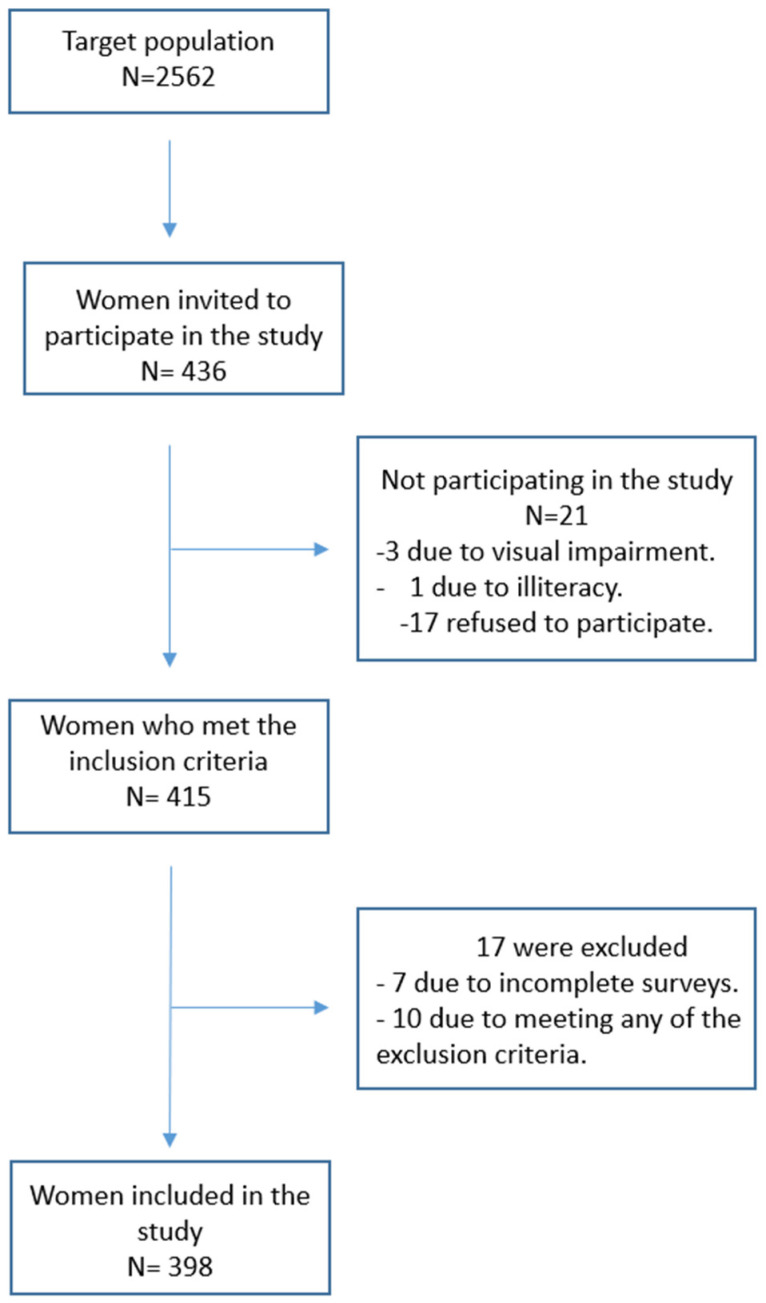
Flowchart of the sampling process.

The sample flowchart is depicted in Figure 2. 

The mean age was 31.25 years, with the youngest participant being 16 years old and the oldest 45 years old, with an SD of 5.34. The description of the rest of the quantitative variables can be seen in Table 1. 

**Figure 3:**
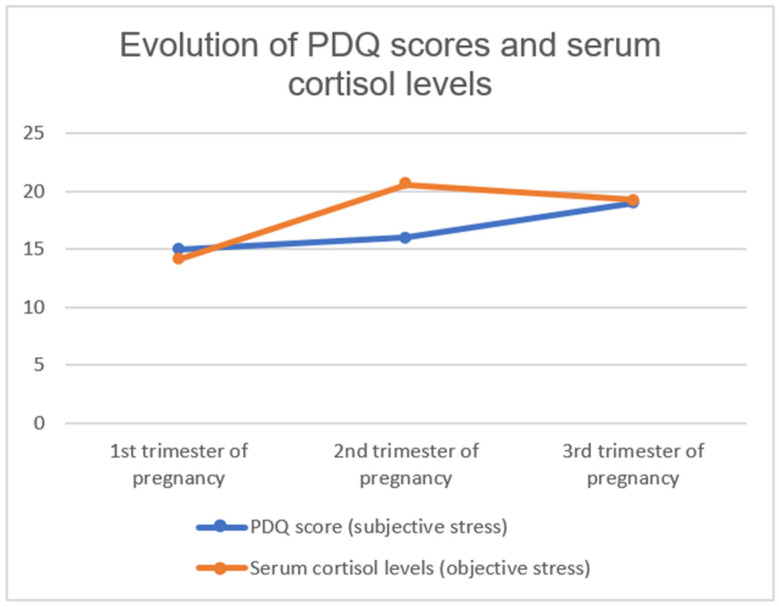
Evolution of PDQ score (subjective stress) and serum cortisol levels (objective stress) across each trimester of pregnancy.

The level of stress, measured both subjectively through the PDQ and objectively through serum cortisol levels, increased as pregnancy progressed. Stress as measured through the PDQ followed a steady increase in each trimester of pregnancy, while serum cortisol levels peaked at their highest in the second trimester. Figure 3 shows how the median values for PDQ scores and serum cortisol levels evolved in each trimester of pregnancy. 

Looking at the descriptive analysis of the qualitative variables, it could be observed that practically half of the sample were married while the other half were single. Being single meant that they lived with their partner, but for whatever reason, they were not officially married. Eighty percent of the sample had a secondary or university education, more than half of them were in active employment, and the socioeconomic level of almost half of the sample (43.2%) was medium-low (>1000 <2000 euros per month).

Most women felt highly supported by their partners, and almost half felt that pregnancy would negatively affect their work situation in a moderate or severe way.

Almost half of the sample attended maternal education classes while the other half did not. Most women opted for epidural analgesia to ease the pain of labour and just over half of the women had a eutocic delivery. About 28% of the women had a caesarean section (Table 2).

### 3.2. Correlational Analysis

Among the most important findings of the bivariate correlations, the following observations were made:

First-trimester serum cortisol levels (objective stress) correlated positively and significantly with PDQ (subjective stress) scores (*p* = 0.04 correlation coefficient 0.1). Similarly, third-trimester serum cortisol levels also correlated positively and significantly with third-trimester PDQ scores (*p* = 0.001 correlation coefficient 0.18). Although these correlations were very weak, they did show significance.

A significant negative correlation was also found between PDQ scores in each trimester and the Apgar test at both minute 1 and minute 5. Thus, the higher the PDQ scores, the lower the Apgar test scores at minutes 1 and 5 of the newborn’s life (*p* = 0.001–0.02 correlation coefficient between 0.12 y 0.17). These correlations, although weak, were still significant (Table 3). 

Second- and third-trimester serum cortisol levels correlated negatively and significantly with birth experience (*p* = 0.01 correlation coefficient 0.13 for the second trimester and *p* = 0.004 correlation coefficient 0.15 for the third trimester). This correlation was also weak, yet significant. Thus, women who had higher levels of serum cortisol in the second and third trimesters had a worse birth experience.

Finally, a strong correlation was found across PDQ scores (subjective stress) between serum cortisol at the second and third trimesters (*p* = 0.001 correlation coefficient 0.93 for the second trimester and 0.86 for the third trimester). The rest of the correlations can be seen in Table 3.

### 3.3. Comparison of Medians and Bivariate Analysis

Statistically significant differences were found when assessing the following variables according to the PDQ (subjective stress) scores in the third trimester of pregnancy: variables preceding stress—employment status, perceived degree of support by the woman during pregnancy, belief that pregnancy may negatively affect her employment status, and attendance of prenatal education classes—and variables following stress—type of delivery and stitches received (Table 4). 

Among the variables preceding stress, housekeepers had higher levels of prenatal distress and, therefore, showed higher levels of subjective stress than those who were unemployed or working (*p* = 0.002). The post hoc test established that statistically significant differences across groups were found between the group of unemployed women and the group of women who were working (*p* = 0.002) and between the group of unemployed women and the group of housekeepers (*p* = 0.01).

Women who perceived a high degree of support from their partner or a close family member had lower stress scores on the PDQ questionnaire and lower levels of prenatal distress compared with those who reported no support or a moderate degree of support. The post hoc test established that statistically significant differences across groups were found between the group of women who felt a high degree of support and the group of women who felt that support was moderate (*p* = 0.002).

Women who believed that pregnancy would not affect their employment status at all scored lower on the PDQ, thus showing lower levels of prenatal distress and, consequently, subjective stress. The higher their distress for this reason, the higher they scored on the PDQ (*p* = 0.003). The post hoc test established that statistically significant differences across groups were found between the group of women who believed that pregnancy would not affect their employment status at all and the group whose distress was moderate (*p* = 0.003) and between the group of women who believed that pregnancy would not affect their employment status at all and the group whose distress was very high (*p* = 0.001).

In terms of attending prenatal education classes, statistically significant differences were also found (*p* = 0.001), with women who did attend scoring lower on the PDQ.

Considering the variables following stress, with regard to the type of delivery once the pregnancy was over, women with a low PDQ score were more likely to have a eutocic delivery. Conversely, those with higher PDQ scores were more likely to have a caesarean section. The associated statistical significance was *p* = 0.001. The post hoc test established that statistically significant differences across groups were found between the group of women who had a eutocic delivery and the group of women who had a caesarean section (*p* = 0.001).

Likewise, looking at the stitches received at delivery, it was observed that women who obtained higher scores on the PDQ, showing greater levels of both subjective stress and prenatal distress, were those who ended up having a caesarean section. The post hoc test established that statistically significant differences across groups were found between the group of women who had a caesarean section and the group of women who did not have any stitches (*p* = 0.001) and between the group of women who had a caesarean section and the group of women who had suffered tearing (*p* = 0.001).

Regarding serum cortisol levels in the third trimester of pregnancy, statistically significant differences were found when relating them to the type of delivery, the stitches received at delivery, and the type of analgesia employed (Table 5).

Women who had a caesarean delivery were found to have higher serum cortisol levels than those who had a vaginal delivery. The associated statistical significance was *p* = 0.001. The post hoc test established that statistically significant differences across groups were found between the group of women who had a eutocic delivery and the group of women who had a caesarean section (*p* = 0.001) and between the group of women who had an assisted delivery and the group of women who had a caesarean section (*p* = 0.001).

When relating serum cortisol levels in the third trimester of pregnancy to the stitches received at delivery, it was observed that women with the lowest cortisol levels ended up with an intact perineum or with some vaginal tearing, followed by those who had an episiotomy. Finally, women with the highest cortisol levels had a caesarean section. The associated statistical significance was *p* = 0.001. The post hoc test established that statistically significant differences across groups were found between the group of women who had a caesarean section and the rest of the groups (*p* = 0.001).

Serum cortisol levels in the third trimester also showed a statistical significance when related to the type of analgesia used for labour. Women with lower serum cortisol levels made less use of analgesia. Women with higher cortisol levels in the third trimester made more use of epidural or spinal analgesia for caesarean sections. The statistical significance was also 0.001, and the post hoc test established that statistically significant differences across groups were found between the group of women who received epidural analgesia and the group of women who received no analgesia (*p* = 0.001) and between the group of women who received epidural analgesia and the group of women who received local analgesia (*p* = 0.001).

Effect sizes were calculated using Hedges’ g and are included in Table 4 and Table 5.

### 3.4. Multivariate Analysis

Two binary logistic regression models were run. In the first (Table 6), the dependent variable used was the PDQ score, which measured women’s perceived stress through prenatal distress. The most interesting finding obtained with this model was that women with higher cortisol levels in the third trimester had a 1.75 times higher probability of having higher levels of subjective stress in this trimester. They also had a 2.14 times higher probability of reporting higher levels of stress in the second trimester and a 1.59 times higher probability in the first trimester.

In the second binary logistic regression model, serum cortisol levels in the third trimester were taken as the dependent variable to measure the stress women were experiencing in an objective way (Table 7). The main finding obtained with this model was that women who had a caesarean delivery were 2.3 times more likely to have higher serum cortisol levels in the third trimester of pregnancy.

## 4. Discussion

Psychological distress during pregnancy refers to the concerns and distress that arise during pregnancy and that can be assessed by means of psychological scales. Stress is something that differs from person to person, so two individuals will respond differently to the same situation [1]. This study provides relevant information on the matter and is the result of research carried out on this life process.

Among the main findings of the present study, subjective stress, as assessed by the PDQ [31], was found to increase progressively. These findings are in line with those found in Diaz’s study [33], which shows that stress in pregnancy largely stems from the anticipation of impending childbirth and the uncertainty of the final outcome. It is natural for pregnant women to have concerns and fears, including fear for themselves and their health during this period and childbirth, as well as fear of pain and death. Fear for the child and the impending uncertainty of the final outcome are the two key factors that make it a stressful event [33].

An increase in cortisol levels was also observed in the second trimester, decreasing slightly in the third trimester to values above those found in the first trimester of gestation. These results are similar to those found in the study by Akinloye, where serum cortisol also increased significantly in the first trimester, peaked in the second trimester, and decreased in the third trimester [34].

It was found that serum cortisol levels (objective stress) correlated with scores on the PDQ (subjective stress). These data are supported by the study by Caparrós, which detailed that the pituitary gland increases in size during pregnancy, thereby increasing cortisol production; in turn, psychological distress correlates with increased cortisol levels as a response to stress, preparing the body to withstand and overcome a stressful stimulus [35]. However, Himes [36] stated that pregnant women’s perception of stress did not correlate with cortisol secretion in the blood or with any impact on the foetus at birth.

It is worth pointing out that second-trimester cortisol analysis coincides with the oral glucose overload test of 50 g of glucose that is performed on the pregnant woman as part of the usual screening for pregnancy control, i.e., the O’Sullivan test. This test is protocolised as follows: in the morning on an empty stomach, the pregnant woman undergoes a blood glucose profile, and then the oral glucose overload is administered. One hour later, all the second-trimester blood tests are taken, including serum cortisol levels. It could be a priori inferred that second-trimester cortisol would be abnormally elevated due to glucose intake prior to blood sampling; whilst this is not fully clear or confirmed, studies have shown a bidirectional relationship between cortisol and food. High cortisol levels increase the brain’s reward system in favour of the consumption of ‘comfort’ foods, which are usually low-quality snacks (high in sugar and fat). It is also known that consumption of high-sugar foods leads to increased cortisol levels [37].

Women who experienced higher subjective levels of stress obtained higher cortisol levels, and in turn, the study indicates that women with higher cortisol levels had worse obstetric outcomes as they were at a higher risk of having a caesarean section at the end of labour. In this regard, Caparrós argued that maternal psychological stress was capable of affecting the levels of different components, such as the stress hormone, cortisol, or neurotransmitters such as dopamine, serotonin, and noradrenaline, involved in the development and functioning of the brain [35,38].

A negative correlation was observed between the Apgar score and third-trimester subjective stress. Although this correlation was weak, it was statistically significant in such a way that when stress increased, the Apgar test score decreased, but the cause was unknown. In line with these findings, Dayan [2] stated that psychological distress during pregnancy was associated with negative consequences for the mother and her offspring; likewise, Serrano [39] noted in another study that the increase in blood corticosteroids, such as cortisol, led to a negative effect on the immunity of the pregnant woman, making her more susceptible to any pathology and affecting the foetus in the same way.

Women who had a worse birth experience were also found to have higher serum cortisol levels in the third trimester. This is consistent with another study, which stated that objective stress significantly influenced the woman’s experience of childbirth. Increased adrenaline release can interfere with the release of endogenous oxytocin, thus hindering the physiological course of labour and inhibiting the production of endogenous opioids. This leads to a fear–tension–pain cycle, which results in a negative experience of childbirth [40].

Women who attended prenatal education classes scored lower on the PDQ, which could indicate lower levels of subjective stress during the third trimester of pregnancy. This led to a better birth experience owing to lower levels of anxiety and better obstetric outcomes. These results are in line with those reported by Dunkel who stated that a positive perception of social support was associated with lower levels of anxiety and postpartum depression in pregnant women, and it has been shown that receiving support from a partner during pregnancy predicts good prenatal mental health [41]. Awad also reported that women who participated in childbirth preparation classes showed lower levels of subjective and physiological stress, most likely due to the information received in these sessions and the interaction with other women in the same situation [42].

Women who had an assisted birth were seen to have higher serum cortisol levels in the third trimester [43], where the type of delivery had a significant influence on cortisol levels, i.e., the higher the cortisol levels, the more dystocic deliveries. Women who believed that pregnancy could negatively affect their employment status showed higher levels of stress than those who did not hold this belief. This was consistent with Aular [44] describing that pregnancy is often seen by companies as a sure reason for absenteeism, poorer physical condition, and occupational aptitude, as well as a reason for illness. This business perspective generates high levels of stress in female workers that adversely impact their health and the health of the foetus.

Among the limitations of the study, it should be noted that the measurement of cortisol is complicated as it depends on many factors, such as the circadian cycle, and, also, because cortisol can be measured in different ways [45]. In an attempt to mitigate this limitation, blood collection was always carried out at the same time of day, between 8 and 9 a.m., following the standardised method at the centre.

Another limitation is the small geographical dispersion of the sample as it was taken from a single study centre. This sample was representative of this limited area, although it should not differ much from other populations with similar socioeconomic and cultural characteristics, such as married marital status, working, intermediate level of education, or average income.

The proximity of southern Spain to northern Morocco should also be noted as a geographical limitation. This means that there is a considerable representation of individuals of Moroccan culture in the study population, which was not taken into account in this research. There are studies that postulate that the process of acculturation has a direct effect on perinatal outcomes [46]. In addition, it is worth noting the high prevalence of women with diabetes as a result of their diet and lifestyle, as well as poorer gestational control due to cultural differences [47]. 

Also, it should be noted that in order to dichotomise the variables in the binary logistic regression models, mean values were used as cutoff points for the cortisol and PDQ variables. Although this approach could be problematic and even lead to a certain misclassification bias, the concept is considered to be pedagogically well understood. The analysis of this study was exploratory and the possibility of a type I error cannot be excluded. Still, 95% confidence intervals (CI) and a significance level of *p* < 0.05 were achieved.

Additionally, among the strengths of this study, validated questionnaires with a high level of reliability were used to measure the study parameters: the Spanish-validated questionnaire (CEQ-E) was used for the childbirth experience and the PQD was used to measure distress.

## 5. Conclusions

High levels of prenatal stress were associated with increased maternal and infant morbidity during pregnancy and childbirth. Women who experienced higher levels of stress in the third trimester of pregnancy were more likely to have poorer obstetric outcomes, with a higher risk of having a caesarean section.

Likewise, high levels of maternal stress directly influenced neonatal morbidity, increasing the risk of lower Apgar scores in the first minute of life.

Women who attended prenatal education classes had lower levels of prenatal stress because, during these sessions, the assisting midwives and health staff provided information to women and their partners, clarifying all doubts and comforting them, thus reducing prenatal distress to a great extent. This contributed to women having a more positive experience of childbirth and better obstetric outcomes. Therefore, it is essential for health systems to implement quality prenatal care and training programmes and to reach all women and their environment.

Given that high cortisol levels were the deciding factor for complicated births and caesarean sections, it would be interesting to further explore the correlation between prenatal stress, prenatal education, and family support, and their direct effects on maternal–foetal morbidity in future research as it may be a good indicator for improving obstetric outcomes. The need to know the levels of stress experienced by pregnant women on the part of the healthcare staff can influence the practice of childbirth and its management. Multidisciplinary intervention is necessary in the healthcare setting for early detection of this type of problem.

## Figures and Tables

**Table 1:** Descriptive study of the quantitative variables.

	N	Median	Range
Number of children	398	0	(0–3)
Number of terminations	398	0	(0–1)
Gestational week at delivery	398	40	(38–42)
Weight of the newborn	398	3300	(2926–3576)
Apgar test score at minute 1	398	9	(9)
Apgar test score at minute 5	398	10	(9–10)
PDQ test score in the 1st trimester of pregnancy	398	15	(11–18)
PDQ test score in the 2nd trimester of pregnancy	398	16	(12–19)
PDQ test score in the 3rd trimester of pregnancy	398	19	(14–25)
Serum cortisol levels in the 1st trimester of pregnancy	398	14.2	(11.6–17.8)
Serum cortisol levels in the 2nd trimester of pregnancy	398	20.6	(17.6–22.9)
Serum cortisol levels in the 3rd trimester of pregnancy	398	19.3	(17.3–21.9)
CEQ-E test score	398	63	(55–71)

**Table 2:** Descriptive study of the qualitative variables.

Variable	Values	Frequency	Percentages
Marital status	Single	196	49.3
	Married	194	48.7
	Divorced	8	2
Education	No studies	8	2
	Primary studies	78	19.6
	Upper secondary studies or vocational training	176	44.2
	University or postgraduate	136	34.2
Employment status	Unemployed	100	25.1
	Working	240	60.3
	Housekeeper	58	14.6
Economic income	<EUR 1000/month	66	16.6
	EUR 1000–2000/month	172	43.2
	EUR 2001–3000/month	108	27.1
	>EUR 3000/month	52	13.1
Supporting person	Nobody	6	1.5
	Partner	346	86.9
	Another relative	46	11.6
Perceived degree of support	No perceived support at all	2	0.5
	Little	16	4
	Moderate	74	18.6
	High	306	76.9
Belief that pregnancy will affect her employment status	It does not affect	152	38.2
	Slightly affects	96	24.1
	Moderately affects	98	24.6
	Seriously affects	52	13.1
Attendance of prenatal education classes	Yes	184	45.7
	No	214	53.8
Analgesia for childbirth	No analgesia	68	17.1
	Local analgesia	50	12.6
	Epidural or spinal analgesia	280	70.4
Type of delivery	Eutocic	208	52.3
	Assisted	78	19.6
	Caesarean	112	28.1
Stitches	No stitches	92	23.1
	Perineal tearing	130	32,7
	Episiotomy	64	16.1
	Inherent to a caesarean section	112	28.1

**Table 3:** Correlational analysis of the different quantitative variables.

		Age	Number of Children	Apgar min 1	Apgar min 5	Birth Experience	Cortisol 1st Trim.	Cortisol 2nd Trim.	Cortisol 3rd Trim.	PDQ 1st Trim.	PDQ 2nd Trim.	PDQ 3rd Trim.
Age	Correlation coeff.		0.21 *	−0.91	−0.06	−0.94	−0.161 *	−0.123 *	−0.04	0.07	0.04	0.1
	Sig. (bilateral)		0.001	0.07	0.25	0.61	0.001	0.01	0.4	0.2	0.48	0.06
Number of children	Correlation coeff.			0.06	0.09	0.168 *	−0.11 *	−0.15 *	−0.08	−0.11 *	−0.12 *	−0.02
	Sig. (bilateral)			0.24	0.07	0.001	0.03	0.003	0.12	0.03	0.01	0.74
Apgar min 1	Correlation coeff.				0.9 *	0,1	0.08	0.03	0.04	−0.12 *	−0.13 *	−0.13 *
	Sig. (bilateral)				0.001	0.05	0.1	0.6	0.43	0.01	0.009	0.01
Apgar min 5	Correlation coeff.					0.05	0.05	0.02	0.05	−0.17 *	−0.16 *	−0.16 *
	Sig. (bilateral)					0.28	0.3	0.69	0.27	0.001	0.001	0.02
Experience	Correlation coeff.						−0.03	−0.13 *	−0.15 *	0.2 *	−0.18 *	−0.15 *
	Sig. (bilateral)						0.58	0.01	0.004	0.001	0.001	0.002
Cortisol 1st trim.	Correlation coeff.							0.3 *	0.08	0.1 *	0.22 *	0.42 *
	Sig. (bilateral)							0.001	0.13	0.04	0.001	0.001
Cortisol 2nd trim.	Correlation coeff.									−0.01	−0.02	−0.01
	Sig. (bilateral)									0.98	0.68	0.84
Cortisol 3rd trim.	Correlation coeff.									0.02	0.04	0.18 *
	Sig. (bilateral)									0.66	0.5	0.001
PDQ 1st trim.	Correlation coeff.										0.93 *	0.86 *
	Sig. (bilateral)										0.001	0.001
PDQ 2nd trim.	Correlation coeff.											0.9 *
	Sig. (bilateral)											0.001

* Spearman’s Rho correlation coefficient.

**Table 4:** Comparison of medians for subjective stress-related variables in the third trimester of pregnancy (PDQ scores).

Variable	Values	N	Median	Range	*p* Value	Hedges’ g
Prior to stress Employment status	Unemployed	100	16	(13–22)	0.002 *	Ref.
	Working	240	20	(15–25)		0.26
	Housekeeper	58	21	(17.5–26)		0.42
Degree of perceived support	No perceived support at all	2	32	(32–32)	0.001 *	1.7
	Little	16	18	(13.23–23.75)		0.1
	Moderate	74	22	(17.75–26)		0.51
	High	306	18	(13.24–24.25)		Ref.
Belief that pregnancy will affect her employment status	It does not affect	152	18	(13–24)	0.003 *	Ref.
	Slightly affects	96	18.5	(13–25)		0.11
	Moderately affects	98	21	(14.75–26)		0.39
	Seriously affects	52	21.5	(17–26)		0.55
Attendance of prenatal education classes Following stress	Yes	184	18	(11–24)	0.001 **	Ref.
	No	214	20	(15–26)		0.36
Type of delivery	Eutocic	208	18	(13–22.75)	0.001 *	Ref.
	Assisted	78	19	(13–26)		0.22
	Caesarean	112	22	(15.25–26.75)		0.5
Stitches	No stitches	92	19	(13–24)	0.002 *	Ref.
	Perineal tearing	130	18	(12.75–22)		0.07
	Episiotomy	64	19.5	(14–25.75)		0.11
	Inherent to a caesarean section	112	22	(15.25–26.75)		0.41

* Kruskall–Wallis test. ** Mann–Whitney U test.

**Table 5:** Comparison of medians for variables related to objective stress in the third trimester of pregnancy (serum cortisol levels).

Variable	Values	N	Median	Range	*p* Value *	Hedges’ g
Type of delivery	Eutocic	208	18.5	(17.3–21.3)	0.001	Ref.
	Assisted	78	18.2	(16.1–21.9)		0.02
	Caesarean	112	21.25	(18.05–23.6)		0.69
Stitches	No stitches	92	18.25	(16.3–21.3)	0.001	Ref.
	Perineal tearing	130	18.1	(16.95–21.22)		0.01
	Episiotomy	64	19.8	(16.27–22.05)		0.21
	Inherent to a caesarean section	112	21.25	(18.05–23.6)		0.69
Analgesia for childbirth	No analgesia	68	17.95	(15.9–20.9)	0.001	Ref.
	Local analgesia	50	18.2	(16.32–21.05)		0.11
	Epidural or spinal analgesia	280	20.55	(17.4–22.28)		0.52

* Kruskall–Wallis test.

**Table 6:** Binary logistic regression model for the PDQ scores dependent variable (subjective stress).

Independent Variables	Sig.	Exp(B)	95% C.I. for EXP(B)	
			Lower	Upper
First-trimester serum cortisol	0.001	0.43	0.34	0.55
Third-trimester serum cortisol	0.001	1.75	1.39	2.2
First-trimester PDQ score	0.001	1.59	1.26	1.99
Second-trimester PDQ score	0.001	2.14	1.61	2.85

Dependent variable: PDQ (subjective stress) score in the third trimester of pregnancy. Independent variables included in the model: employment status, degree of affectation to employment status, degree of perceived support, attendance of prenatal education classes, first-trimester PDQ score, second-trimester PDQ score, first-trimester serum cortisol level, type of delivery, stitches received, the weight of the newborn, and birth experience. Hosmer and Lemeshow test chi-squared: 16.62 (*p* = 0.003) Nagelkerke’s R^2^ = 0.9.

**Table 7:** Binary logistic regression model for the serum cortisol levels dependent variable.

Independent Variables	Sig.	Exp(B)	95% C.I. for EXP(B)	
			Lower	Upper
Weight of newborn	0.004	0.99	0.99	1
Third-trimester PDQ score	0.001	1.06	1.03	1.09
Type of delivery: eutocic	0.002	Ref.		
Type of delivery: assisted	0.66	0.88	0.51	1.53
Type of delivery: caesarean section	0.001	2.3	1.39	3.81
Degree of support: no	0.01	Ref.		
Degree of support: low	0.9	0	0	0
Degree of support: moderate	0.03	3.51	1.08	11.38
Degree of support: high	0.01	0.49	0.28	0.88

Dependent variable: serum cortisol levels in the third trimester of pregnancy. Independent variables included in the model: weight of the newborn, birth experience, subjective stress in the third trimester of pregnancy, type of delivery, perceived degree of support, and analgesia for childbirth. Hosmer and Lemeshow test chi-squared 29.77 (*p* = 0.001). Nagelkerke R^2^ = 0.46.

## Data Availability

Data are available upon a reasonable request made to the corresponding authors.
